# Di(2-ethylhexyl) phthalate-induced toxicity and peroxisome proliferator-activated receptor alpha: a review

**DOI:** 10.1186/s12199-019-0802-z

**Published:** 2019-07-06

**Authors:** Yuki Ito, Michihiro Kamijima, Tamie Nakajima

**Affiliations:** 1 Department of Occupational and Environmental Health, Nagoya City University Graduate School of Medical Sciences, Nagoya, 467-8601 Japan; 2 College of Life and Health Sciences, Chubu University, 1200 Matsumoto-cho, Kasugai, Aichi 487-8501 Japan

**Keywords:** Di(2-ethylhexyl) phthalate, Carcinogenesis, Reproductive toxicity, Developmental toxicity, Science policy

## Abstract

The plasticizer di(2-ethylhexyl) phthalate (DEHP) has been widely used in the manufacture of polyvinyl chloride-containing products such as medical and consumer goods. Humans can easily be exposed to it because DEHP is ubiquitous in the environment. Recent research on the adverse effects of DEHP has focused on reproductive and developmental toxicity in rodents and/or humans. DEHP is a representative of the peroxisome proliferators. Therefore, peroxisome proliferator-activated receptor alpha (PPARα)-dependent pathways are the expected mode of action of several kinds of DEHP-induced toxicities. In this review, we summarize DEHP kinetics and its mechanisms of carcinogenicity and reproductive and developmental toxicity in relation to PPARα. Additionally, we give an overview of the impacts of science policy on exposure sources.

## Introduction

Di(2-ethylhexyl) phthalate (DEHP, CAS No. 117-81-7) is the diester of phthalic acid (PA) and the branched-chain 2-ethyl-1-hexanol (2-EH). It is colorless, viscous, and soluble in lipophilic liquid. DEHP is the most commonly used plasticizer that makes plastic more flexible and elastic. It is used globally, and its shipping volume of 103,499 t accounted for approximately 50% of that of total phthalates in Japan in 2018 [1]. DEHP is widely used in various polyvinyl chloride (PVC) products such as plastic sheets, artificial leather, wire coverings, agricultural vinyl films, pastes, coating materials, medical products, and adhesive agents [1]. Because DEHP is contained in plastic materials without chemical bond, it can easily diffuse into the environment under high temperatures or during contact with hydrophobic materials, leading to ubiquitous environmental contamination. It can also be detected in air due to its use in many products, although little DEHP is intrinsically present in the air because it does not evaporate easily [2]. Moreover, DEHP binds to dust particles in air, binds strongly to soil, and dissolves very slowly in groundwater [3].

Given its omnipresence, there is a high likelihood that the general population will be exposed to DEHP. Recently, increasing attention has focused on the risks of DEHP, particularly in the fields in carcinogenesis and child health. In this review, we summarize the kinetics of DEHP and its mechanisms of carcinogenicity and reproductive and developmental toxicity in relation to nuclear receptor peroxisome proliferator-activated receptor alpha (PPARα), which has been implicated in the mode of action of several types of DEHP-induced toxicities. Additionally, we provide an overview of the impacts of science policy on the sources of exposure.

## Kinetics of DEHP

When DEHP is absorbed by the body, it is first metabolized (mainly by lipase) to mono(2-ethylhexyl) phthalate (MEHP) and 2-EH. A part of MEHP is then conjugated with UDP-glucuronide by UDP-glucuronosyltransferase (UGT) and excreted in the urine. The remaining MEHP is excreted directly in the urine or is oxidized by cytochrome P450 (CYP) 4A then further oxidized by alcohol dehydrogenase (ADH) or aldehyde dehydrogenase (ALDH) to dicarboxylic acid or ketones. 2-EH is metabolized primarily to 2-ethylhexanoic acid (2-EHA) via 2-ethyl-1-hexanal by the catalytic action of ADH and ALDH [4–6]. In these enzymes, lipase activity shows prominent differences among mice, rats, and marmosets. An almost 160–240-fold difference has been reported, caused by the lower affinity of DEHP for lipase in marmosets and the significant differences in the *Vmax/Km* values of lipase for DEHP between the highest (in mice) and the lowest (in marmosets) activity [7]. These results correspond to the fact that lipase activity in humans is lower than that in mice, with only one seventh of the *Vmax/Km* value of lipase for DEHP [8]. In concordance with species differences in lipase activity, hepatic MEHP levels were significantly higher in mice and rats than in marmosets [9]. After DEHP treatment, species differences were also detected in the induction of peroxisome proliferator-activated receptor (PPAR) alpha-mediated enzymes, especially in peroxisomal enzymes such as 3-ketoacyl-CoA thiolase A and peroxisomal bifunctional protein [9, 10]. This was partly due to the different constitutive levels of PPARα and different formation levels of MEHP resulting from the hydrolysis of DEHP.

**Fig. 1:**
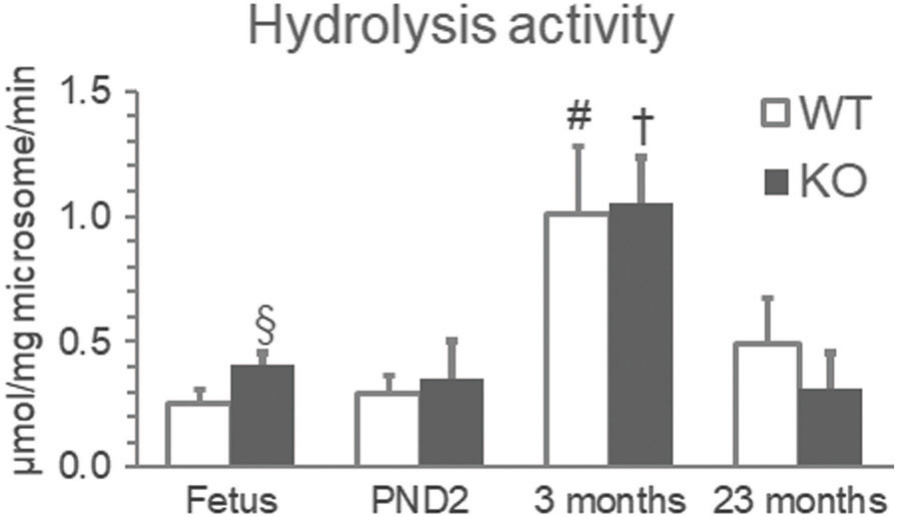
Age differences in hydrolysis activity of DEHP in the hepatic microsome. The livers were removed from fetuses at GD 28 and PND2 and at 3 and 23 months of age. Analytical methods were described elsewhere [11]. Data indicates the mean ± standard deviation. The white and black columns indicate WT and KO, respectively. ^#^Significantly different (*p* < 0.05) from those of other ages in WT. ^†^Significantly different from those of other ages in KO. ^§^Significantly different from that in WT. WT, wild-type mice; KO, *Ppar*α-null mice. White and black bars indicate WT and KO, respectively. *n* = 4–9

DEHP-hydrolyzing capacity depends on age and gestation. Microsomal lipase activities in the livers collected from PPARα wild-type (WT) fetuses on gestational day (GD) 18, pups on postnatal day 2 (PND2), and 23-month-old mice were approximately one quarter, one third, and one half of that in 3-month-old mice, respectively (Fig. 1). Intriguingly, lipase activity was 1.8-fold higher in pregnant dams than in postpartum ones, although no differences were observed in the activity between fetuses and pups [11]. In concordance with their mother’s lipase activity, MEHP concentrations were from 1.5 to 1.7-fold higher in fetuses and their mother dams than in PND2 pups and their mother dams, respectively, after exposure to 0.05% DEHP [11], suggesting that MEHP levels in the blood of the offspring (either fetus or pup) might be related to their mothers’ hepatic lipase activity. Exposure via the placenta in fetuses and milk in pups as well as the production of MEHP in the offspring was implied. UGT activity in fetuses was half of that in pups [11], suggesting that the internal dosage of an active metabolite, MEHP, was likely to be higher in fetuses than in pups. In humans, inter-individual differences in the metabolism of DEHP have been reported, which were greater than the species differences between mice and humans [12]. Inter-individual differences in DEHP metabolism in humans may therefore be more crucial than species differences and thus more important in risk assessment.

**Fig. 2:**
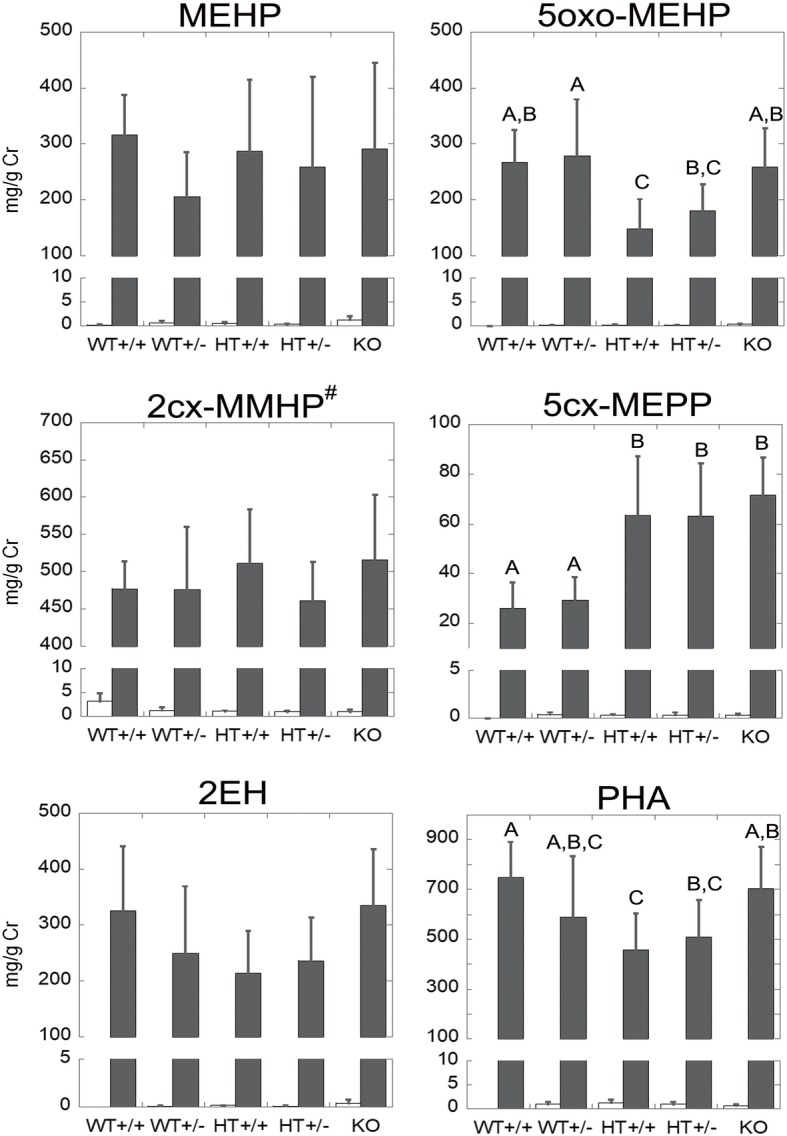
DEHP metabolite concentrations excreted in 24-h urine. Data indicates the mean ± standard deviation. Each letter represents grouping, and different letters indicate significant differences (*p* < 0.05), i.e., there were significant differences between the groups A and B, A and C, and B and C. White and black columns indicate urinary metabolites in 20-week-old male mice fed with control and 0.1% DEHP-containing food for 8 weeks, respectively. These metabolites were measured by gas chromatography-mass spectrometry in Kansai Technical Center for Occupational Medicine, Japan. The detailed methods were described elsewhere [12]. ^#^The values of 2cx-MMHP contain a part of PA. WT, wild-type mice; KO, *Ppar*α-null mice; HT, PPARα-humanized^Tet-Off^ mice; +/+, homozygous; +/−, heterozygous; MEHP, mono(2-ethylhexyl)phthalate; 5oxo-MEHP, mono(2-ethyl-5-oxohexyl)phthalate; 2cx-MMHP, mono[2-(carboxymethyl)hexyl]phthalate; 5cx-MEPP, mono(2-ethyl-5-carboxypentyl)phthalate; 2EH, 2-ethyl-1-hexanol; PHA, phthalic anhydride; Cr, urinary creatinine. *n* = 5–7

Possible involvement of PPARα in DEHP metabolism was suggested in our study (Fig. 2). Excreted amounts of metabolites such as mono(2-ethyl-5-oxohexyl)phthalate (5oxo-MEHP), mono(2-ethyl-5-carboxypentyl)phthalate (5cx-MEPP), and phthalic anhydride (PHA) in 24-h urine differed among WT, Pparα-null (KO), and PPARα-humanized^Tet-Off^ (HT) mice with KO mouse backgrounds after 0.1% DEHP exposure for 8 weeks were shown in Fig. 2. The amounts of 5cx-MEPP were higher in HT and KO mice than in WT mice. The expression of PPARα in HT mice was comparable [13] or higher [14] than that in WT mice. This suggests that the functional differences of PPARα between humans and mice affect the metabolite composition profile, and PPARα might be involved in the pathway of 5cx-MEPP production. Interestingly, 5oxo-MEHP and 5cx-MEPP levels were more dominant in humans than in mice [12]. The relatively weak function of human PPARα compared with that in rodents [10, 14, 15] presumably promotes 5cx-MEPP production via enhancing the activity of the enzyme that catalyzes the production of this metabolite. In other words, if the PPARα function is weak or absent, the enzyme involved in 5cx-MEPP production might be activated.

## PPARα agonist

**Fig. 3:**
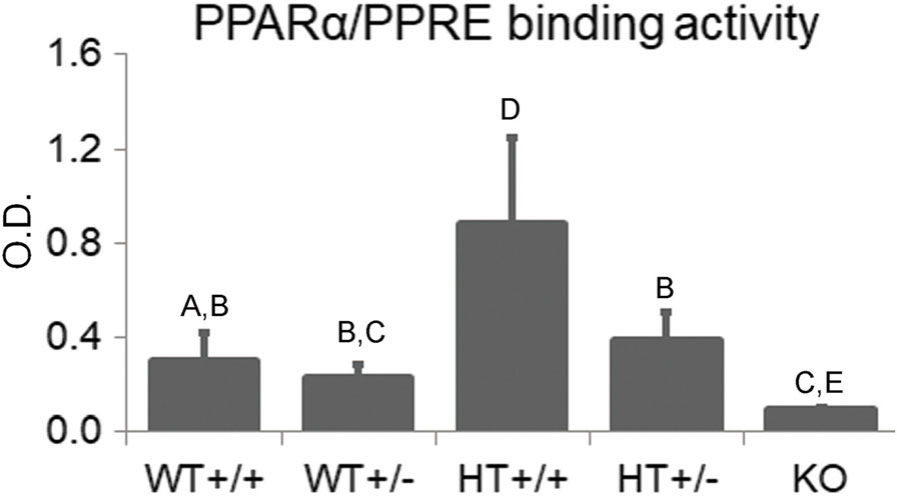
PPARα/PPRE binding activity in the livers from WT, KO, and HT mice. Data indicates the mean ± standard deviation. Each letter represents grouping, and different letters indicate significant differences (*p* < 0.05), i.e., there were significant differences between the groups A and B, A and C, A and D, A and E, B and C, B and D, B and E, C and D, C and E, and D and E. The activity was measured using a PPARα transcription factor assay kit (Cayman Chemical., Ann Arbor, USA) in hepatic nuclear fraction extracted using a CelLytic^TM^ NuCLEAR^TM^ Extraction Kit (SIGMA, Tokyo, Japan). The livers were removed from the respective 28-week-old male mice fed with a control diet and stored at − 80 °C until use. WT, wild-type mice; KO, *Ppar*α-null mice; HT, *hPPARα* mice; +/+, homozygous; +/−, heterozygous. *n* = 6–7

PPARs are members of the nuclear hormone receptor superfamily and consist of three subunits, PPARα, PPARβ/δ, and PPARγ [16]. One of three isoforms, PPARα is mainly expressed in organs that are critical in fatty acid catabolism, such as the liver, heart, and kidneys [17]. Thus, this nuclear receptor is primarily involved in the regulation of fatty acid metabolism. In addition to this function, PPARα also has various other functions including the promotion of gluconeogenesis, lipogenesis, ketogenesis, and anti-inflammatory effects [18]. The mono- and dicarboxylic acid metabolites of DEHP, not DEHP itself, act as ligands for PPARα and PPARγ [19] and have potentially adverse effects on the liver, kidneys, heart, and reproductive organs. After ligand binding, PPAR**/**retinoid X receptor heterodimers bind to specific DNA sequences called PPAR response elements **(**PPREs) in the nucleus and exert various biological functions. Therefore, DEHP potentially alters these functions. In addition, it is presumed that the binding activity of the PPARα/PPRE also depends on species-specific PPARα function or expression levels (Fig. 3).

## Toxicity

DEHP has been shown to cause cancer, reproductive, developmental, nerve, and immune toxicities, and endocrine disruption effects in rodents [2]. Of these toxicities, we focus on the carcinogenicity and reproductive and developmental toxicities.

### Carcinogenicity

Among DEHP-induced toxicities [20–22], hepatic carcinogenicity was the most important issue reported in the past three decades. The cancer risk assessments conducted by many different regulatory authorities have changed over time with the advent of detailed mechanistic information on the involvement of PPARα in the non-genotoxic carcinogenic process, in which metabolites of DEHP activate PPARα signaling as mentioned above. In 1992, based on the hepatocarcinogenesis in rodents (predominantly from National Toxicology Program (NTP) studies) [20, 21, 23], the US Environmental Protection Agency (EPA) and then the International Agency for Research on Cancer (IARC) classified DEHP in categories B2 and 2B, respectively [24, 25]. On this basis, the Japan Society for Occupational Health classified DEHP in category 2B “possibly carcinogenic to humans” [26]. Much later, Doull et al. [27] proposed that the liver tumors were due to PPAR activation and that this mechanism was not relevant for humans and should not be used in human risk assessments. Thus, given the limited available literature on the potential toxic effects of DEHP following human exposure, this mechanistic body of work resulted in the delisting of DEHP as a potential carcinogen (i.e., category 3) by the IARC [28] and the European Union [29]. This decision reflected the absence of data showing effects on peroxisome proliferation in human hepatocyte cultures and non-human primate livers [30, 31]. However, the mechanisms behind the effects of DEHP on PPARα and its subsequent carcinogenicity remained controversial. The Japan Society for Occupational Health did not change the classification. KO mice exposed to DEHP for 22 months exhibited more liver tumors than WT mice [32], and the presumed mechanism differed between the WT and KO mice [33]. Moreover, carcinogenesis was observed in areas other than the livers, such as the pancreas and testis of DEHP-treated Sprague-Dawley rats [34]. Pancreatic acinar adenomas have also been reported as treatment-related findings in chronic studies in F344 rats [35]. These data indicate that DEHP cancer risks should be evaluated not only by focusing on the liver but also on other organs.

As mentioned above, although the expression of human PPAR is similar to that in rodents [13], it is less active against carcinogenesis than that in rodents [10, 14, 15]. However, factors other than PPAR have been suggested to be involved in DEHP hepatocarcinogenesis [36–40]. Some studies pointed out the involvement of non-PPARα pathways as well as PPARα-dependent pathways in DEHP-induced carcinogenicity [32, 41–43]. Of them, the activation of nuclear factor kappa B (NFκB) and other nuclear receptors such as constitutive androstane receptor (CAR) was thought to be possible molecular targets. PPARα exhibits anti-inflammatory activity in WT mice since it represses NFκB. The study using KO mice exposed to DEHP for 22 months showed a dose-dependent increase of NFκB and elevated plasma alanine aminotransferase in KO mice with hepatic tumors [32]. Chronic inflammation promotes genetic and epigenetic aberrations in tumor pathogeneses [44]. A recent paper implied that two modes of action (MOA) were involved in the DEHP-induced tumors in KO mice: steatosis, accumulated in the aged KO mice, played a role in the onset of inflammation in which inflammatory molecules such as NFκB were involved, or increased activation of CAR-related signaling contributed to liver tumors via cell proliferation and foci formation [45]. Dietary DEHP exposure for 2 weeks activated CAR much more strongly in KO mice than in WT mice [46]. It is not known if the weak activation of CAR in WT mice leads to key events downstream other than a weak activation of some CAR-dependent genes, similar to in KO mice, in addition to PPARα-dependent MOA [45].

Although little epidemiological evidence supports the association between DEHP and liver cancer in humans, the IARC concluded that “the human relevance of the molecular events leading to DEHP-induced cancer in several target tissues (e.g., the liver and testis) in rats or mice could not be ruled out” and decided to upgrade DEHP to group 2B in 2012 [47]. At present, the expected key events in DEHP-induced hepatocarcinogenesis are as follows: (1) PPARα activation, (2) alteration in cell growth pathways, (3) alteration in hepatocyte growth including the effects on proliferation and apoptosis, and (4) clonal expansion of preneoplastic-initiated hepatocytes, which (5) leads to the increases in hepatocellular adenomas and carcinomas [45]. NFκB is thought to be a modulating factor that could modulate the dose-response behavior, the probability of inducing one or more key events, or the adverse outcome [45].

### Reproductive and developmental toxicity

For many years, DEHP was suspected to have reproductive toxicity in mice and in in vitro studies [21, 48–50], but without being classified, and in the 1990s, DEHP was also suspected to disrupt the estrogen levels in in vitro studies and female adult rats [51–53]. However, studies have supported the hypothesis that DEHP can harm the reproductive system due to an anti-androgenic effect [54], and these findings have placed DEHP on the EU list of dangerous substances, where it is now classified as a substance which may be regarded to impair fertility and cause developmental toxicity in humans (category 2) [55]. The Japan Society for Occupational Health classified DEHP in group 1 “substances known to cause reproductive toxicity in humans” [56]. In animal studies, DEHP exposure induces testicular morphological changes (especially in Leydig and Sertoli cells), reproductive tract developmental anomalies, sperm damage, disruption of endocrine hormones, changes in the birth sizes of offspring, and anogenital distance [54, 57, 58]. Some epidemiological studies have reported a negative association between DEHP exposure and steroid hormones in the cord blood. In Japan, maternal blood MEHP levels were associated with reduced levels of testosterone (T)/estradiol (E2), progesterone (P4), inhibin B, and insulin-like factor 3 in male cord blood [59], with reduced cortisol and cortisone levels and a lower glucocorticoid/adrenal androgen ratio, but increased dehydroepiandrostenedione (DHEA) levels and a higher DHEA/androstenedione ratio [60]. In Taiwan, maternal urinary levels of DEHP metabolites were negatively correlated with the free T and T/E2 levels in the cord blood [61]. Child-only or both maternal and child DEHP metabolite levels were associated with decreased levels of free T in males or decreased levels of P4 in females, respectively [62]. Additionally, some epidemiological studies suggest an inverse association between DEHP exposure and neurodevelopmental effects [63–65].

In utero exposure to DEHP caused maternal malnutrition, such as PPARα-dependent decreases in serum triglycerides and essential fatty acids. However, these findings were not observed in the offspring in mice. While serum glucose levels in the pups decreased PPARα dependently, such changes were not observed in their dams [66–71]. However, this phenomenon has not been well-investigated; thus, it remains unknown whether unbalanced nutrition caused by DEHP in utero and during infancy is followed by an increased risk of lifestyle diseases. An epidemiological study also reported that DEHP suppressed leptin concentrations via PPARα in offspring [72], resulting in increased food consumption after weaning [60]. The impacts of DEHP exposure on offspring in their later lives should be clarified.

Regarding the reproductive toxicity, various mechanisms, including anti-androgenic effects, have been proposed but not yet proven. The mechanism of the developmental toxicity of DEHP is also still unclear, although the involvement of PPARα has been suggested [70, 73]. The existence of PPARα-independent pathways in the reproductive toxicity of DEHP was reported at higher dosages [74, 75].

## Exposure sources and regulation

Reportedly, estimated daily DEHP intake was 87–89% from food, 9.5–9.7% from indoor air, and 1.4–2.0% from drinking water in a Canadian population [47], and the intake from ambient air and soil was considerably lower. Thus, the general population is mainly exposed to DEHP via food, particularly fatty foods such as milk, including breast milk. Fish, fats, oils, and freeze-dried foods can also be highly contaminated with DEHP [76].

With regard to food packaging, the use of DEHP in the material that comes into contact with food has already been restricted under Commission Directive 2007/19/EC of March 30, 2007, amending Directive 2002/72/EC relating to plastic materials and articles likely to come into contact with food [77]. In Japan, the use of DEHP in PVC materials used for the packaging of foods containing edible fats and oils is avoided to prevent its migration into food. Furthermore, its use is prohibited in the manufacture of toys [78]. In 2008, as a measure to reduce exposure and protect children’s health, the US Congress passed the Consumer Product Safety Improvement Act, which includes a federal ban on phthalates in toys and child products. This latest legislation prohibits the use of eight phthalates in the manufacture of children’s products [79]. Similarly, DEHP and other phthalates are already restricted through entry 51 of Annex XVII to Regulation (EC) No 1907/2006 of the European Parliament and of the European Council, which legislates that toys containing several phthalates, including DEHP, with a cumulative concentration greater than 0.1% by weight of the plasticized material cannot be placed on the EU market [80]. These restrictions also apply to the cables or spare parts used for the repair, reuse, updating of functionalities, or upgrading of the capacity of electrical and electronic equipment placed on the market after July 22, 2019, and also to the medical devices, including in vitro medical devices, as well as monitoring and control instruments, including industrial monitoring and control instruments, placed on the market after July 22, 2021 [81].

The US Food and Drug Administration (FDA) believes that the greatest concern is for very young male infants who are critically ill and have prolonged exposure to multiple devices containing DEHP [82]. Additionally, the reproductive development of male fetuses (through exposure to their mothers) and peripubertal males would also be at risk. The NTP has reached a similar conclusion [83]. In contrast, there is a little concern for adults receiving intravenous solutions or undergoing peritoneal dialysis, who were previously thought to be a high-risk group.

So far, a number of government agencies and expert panels in Japan, the USA, Canada, and Europe, have reviewed the safety of DEHP in medical devices and toys. Each of these agencies and expert panels has found that DEHP exposure from some medical procedures may pose a risk to the patients’ health and recommended restricting the use of phthalates, including DEHP, in medical devices (Ministry of Health, Labour and Welfare in Japan; European Directive 2007/47/CE; FDA Safety Assessment and a Public Health Notification; Health Canada expert advisory panel on DEHP in medical devices). Medical device manufacturers were therefore forced to quickly find alternatives to DEHP to maintain the elasticity of PVC nutrition tubes, infusion sets, and hemodialysis lines. Several replacement plasticizers, the so-called alternative to DEHP plasticizers such as tri-octyl trimellitate (TOTM), 1,2-cyclohexane dicarboxylic acid diisononylester (DINCH), and di(2-ethylhexyl)cyclohexan-1,4-dicarboxylate (DEHCH), are currently incorporated in modern medical devices [84–86].

**Fig. 4:**
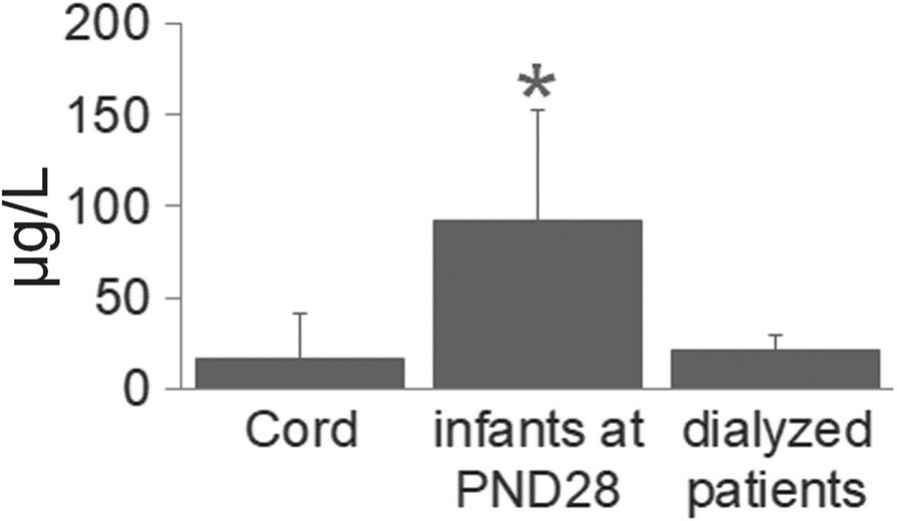
Serum MEHP concentrations in the cord and corresponding PND28 infant in NICU (*n* = 49) and dialyzed patients (*n* = 110). PVC-free tubes were used for feeding the infants in NICU [87, 88]. The serum from the dialyzed patients was collected before a hemodialysis session using plasticizer-free tubes with written informed consent. Data for the serum MEHP concentrations after the hemodialysis session have been presented in our previous paper [89]. The analytical methods have been described elsewhere [7]. *p < 0.05 compared with the other groups

In 2002, the Ministry of Health, Labour and Welfare in Japan recommended the immediate precautionary replacement of all DEHP-softened PVC medical devices in feeding tubes for premature infants and newborns and in dialyzer blood circuits with alternative products. In addition, it was also mentioned that DEHP exposure from transfusion tubes and blood oxygenator tubes should also be minimized when alternatives are available. After this admonition, the changes in MEHP levels between the cord serum and the serum of the 1-month-old newborns in a neonatal intensive care unit (NICU) were reduced significantly by the replacement of PVC feeding tubes with PVC-free feeding tubes (pre-admonition, from 15 μg/L (median) in the cord blood to 185 μg/L in the sera at aged 28 days; post-replacement, from 10 μg/L in the cord blood to 79 μg/L in the sera at aged 28 days) [87, 88]. In 2010, we examined the extraction tests using the medical tubes running in a certain hospital. No DEHP was detected in the feeding tubes for neonates or the transfusing tubes for dialyzed patients, some of which contains TOTM instead of DEHP, although DEHP was detected in blood oxygenator tubes. Importantly, in some cases, when difficulties arose while inserting the tubes, the tubes containing DEHP were used for the neonates [85]. Even when using PVC-free tubes, serum MEHP levels in infants in the NICU were higher than those in the dialyzed patients before a hemodialysis session (Fig. 4). A possible causal factor responsible for the high serum levels of MEHP in infants might be the low activity of UGT in young children [90]. In addition to the medical devices, DEHP contamination in packed food and bottled water was reported, but at considerably lower levels than those putting patients with chronic illnesses at risk [91].

## Recent topics

Many studies show carcinogenicity and reproductive and developmental toxicity in rodents, but human data is limited. A food scandal occurred in Taiwan in 2011 because DEHP had been intentionally used in food products [92]. Further longitudinal epidemiological data will contribute to the risk assessments of DEHP in humans [93].

World production of plastic containing polyethylene (PE), polypropylene, PVC, polystyrene, polyamide, chlorinated PE, and chlorosulfonated PE increased drastically in the last two decades [94], while that in Japan, decreased [95]. Recently, large amounts of these plastics end up in the ocean as so-called microplastics. The microplastic-associated organic plastic additives contain phthalates including DEHP [96]. For all organisms, the combined intake from food and water was the main route of exposure to DEHP with a negligible input from plastic, while a very small increase in the internal concentrations of DEHP was observed in benthic invertebrates [97].

## Conclusions

In this review, we summarized the mechanisms of carcinogenicity and reproductive and developmental toxicity in relation to PPARα. Additionally, we gave an overview of the impacts of science policy on the exposure sources. DEHP use has been restricted because of the potential adverse effects, and DEHP exposure levels in people at higher risk have thereby certainly been reduced.

## Data Availability

Please contact the authors for data requests.

## Abbreviations

+/−: Heterozygous
+/+: Homozygous
2cx-MMHP: Mono[2-(carboxymethyl)hexyl]phthalate
2-EH: 2-Ethyl-1-hexanol
2-EHA: 2-Ethylhexanoic acid
5cx-MEPP: Mono(2-ethyl-5-carboxypentyl)phthalate
5oxo-MEHP: Mono(2-ethyl-5-oxohexyl)phthalate
ADH: Alcohol dehydrogenase
ALDH: Aldehyde dehydrogenase
CAR: Constitute androstane receptor
Cr: Urinary creatinine
CYP: Cytochrome P450
DEHCH: Di(2-ethylhexyl)cyclohexan-1,4-dicarboxylate
DEHP: Di(2-ethylhexyl) phthalate
DHEA: Dehydroepiandrostenedione
DINCH: 1,2-Cyclohexane dicarboxylic acid diisononylester
E2: Estradiol
FDA: Food and Drug Administration
GD: Gestational day
HT: PPARα-humanized^Tet-Off^ mice
IARC: International Agency for Research on Cancer
KO: *Ppar*α-null mice
MEHP: Mono(2-ethylhexyl) phthalate
NFκB: Nuclear factor kappa B
NICU: Neonatal intensive care unit
NTP: National Toxicology Program
P4: Progesterone
PE: Polyethylene
PHA: Phthalic anhydride
PND: Postnatal day
PPAR: Peroxisome proliferator-activated receptor
PVC: Polyvinyl chloride
T: Testosterone
TOTM: Tri-octyl trimellitate
UGT: UDP-glucuronosyltransferase
WT: Wild-type mice
